# Routine versus standardized blood pressure measurements: Agreement and association with cardiovascular disease

**DOI:** 10.1111/joim.70164

**Published:** 2026-09-24

**Authors:** Olivia Gästgivars, Joachim Ögren, Bo Carlberg, Mattias Brunström

**Affiliations:** ^1^ Department of Public Health and Clinical Medicine Umeå University Umeå Sweden; ^2^ Department of Public Health and Clinical Medicine Umeå University Östersund Sweden

**Keywords:** blood pressure determination, cardiovascular diseases, hypertension, primary health care

## Abstract

**Background:**

Recommendations for blood pressure (BP) measurement are poorly followed in clinical practice, possibly leading to inaccurate BP estimates.

**Objectives:**

To assess the agreement between routine and standardized BPs, and the association between BP level and cardiovascular disease (CVD) for each measurement type.

**Methods:**

Standardized BPs were recorded in a screening program for middle‐aged people during 1992–2012 in Sweden. Intraindividual differences between standardized BP and routine BP from primary care were calculated. Association between BP level and composite CVD, and population‐attributable fraction (PAF) explained by BP, were determined through adjusted Cox regression for each measurement type.

**Results:**

In 9567 participants, routine BPs were on average 4.0/1.7 mmHg higher than standardized BPs. Routine measurements frequently misclassified people into higher BP categories; 16% more had hypertension (≥130/80 mmHg), and 50% less had normal BP (<120/80 mmHg), compared with standardized measurements. Analysis of 19,720 participants found a stronger association between BP level and CVD for standardized than for routine measurements, with increased risk from ≥120/80 and ≥140/90 mmHg, respectively. Adjusted hazard ratio for ≥140/90 mmHg compared to <120/80 mmHg was 2.11 (95% confidence interval 1.77–2.51) for standardized BPs and 1.43 (1.15–1.77) for routine BPs. BP ≥120/80 mmHg explained 41.8% (32.0–50.1) of CVD events with standardized measurements and 19.3% (1.5–33.8) with routine measurements.

**Conclusion:**

Routine BPs deviate from standardized BPs, resulting in significant misclassification and attenuated association with CVD, reducing the PAF for BP to less than half. Our findings emphasize the need for accurate BP measurement in clinical practice.

## Graphical abstract

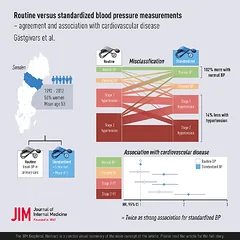

## Introduction

The association between blood pressure (BP) level and cardiovascular disease (CVD), and the beneficial effects of BP‐lowering, have been established in trials where BP was measured in a standardized manner, by trained staff following strict protocols [1, 2, 3]. Recommendations in guidelines mimic such protocols to minimize systematic and random errors in clinical practice [4, 5, 6, 7]. The recommended procedure is often poorly followed, resulting in poor reproducibility and overestimated BP values compared to measurements performed within a research setting [8, 9, 10, 11]. More recently, these findings were supported by BPs from the Systolic Blood Pressure Intervention Trial (SPRINT), which were shown to be lower compared to BPs from electronic health records [12].

Poor adherence to BP measurement recommendations may impair the applicability of trial findings and implementation of guideline recommendations in clinical practice. If routine measurements tend to overestimate BP levels, patients may be overtreated. This is particularly important in the light of lowered treatment thresholds and current discrepancies in the definition of hypertension between guidelines [4, 5, 6]. Further, the predictive value of BPs and the expected treatment benefit may be diminished if routine measurements are subject to random errors and variability between measurements. The association with CVD outcomes is yet to be compared between routine clinical BPs and measurements following standardized procedures.

The overall aim of this study was to investigate the validity and reliability of routine clinic BPs compared with standardized measurements. The objectives were to assess the agreement between routine and standardized BPs, the impact of measurement type on classification into BP categories, differences in the association between BP level and risk for CVD between routine and standardized BPs, and the fraction of CVD explained by uncontrolled BP according to measurement type.

## Methods

### Material and study design

This was a retrospective cohort study, where data from Swedish national registers and local databases were linked using the unique civic number of each individual. The study follows the Strengthening the Reporting of Observational Studies in Epidemiology guidelines [13]. The study was approved by the Swedish Ethical Review Authority (dnr 2020–04149).

The study took place in Västerbotten County in Sweden, in a primary care setting. Standardized BP recordings were gathered from the Västerbotten Intervention Programme (VIP). Routine BP recordings were measured in any clinical situation in primary care without specific instruction and were collected from the Swedish Stroke Prevention Study (SSPS). Data were complemented with diagnoses from the Swedish National Patient Register (NPR) and the Swedish Cause of Death Register, and information on socioeconomic factors and migration status from Statistics Sweden. The temporal overlap of the registers is illustrated in Fig. S1.

VIP is a health intervention program in Västerbotten County, ongoing since 1985, designed to invite all residents for a health examination in primary care in the years they turn 40, 50, and 60. Cardiovascular risk factors are assessed by blood samples, questionnaires, and clinical examination, performed by a nurse according to a detailed manual, forming the VIP database but also documented in the medical record to enable follow‐up [14]. BPs in VIP were measured by trained nurses according to a prespecified protocol. BP was measured after 5 min of rest, once in the supine position until August 2009, and twice in the seated position from September 2009. During the early period, manual sphygmomanometers were used with the auscultatory method. Validated and calibrated automated devices were increasingly used from 2000 onward.

SSPS was an intervention study aiming to improve BP control in Västerbotten County [15]. To assess its effects, all BP recordings from primary care electronic medical records from 1992/1993 to December 2011 were extracted by a validated script. The BP measurements were performed by different staff categories, defined as “doctor” or “other” (further unspecified, but most commonly a nurse). SSPS also contains information on hypertension and diabetes diagnoses.

NPR contains diagnosis codes from all hospital admissions since 1987, with high validity for diagnoses of heart failure (HF), stroke, and myocardial infarction (MI) (positive predictive values and sensitivity ranging from 80% to 100%) [16, 17]. In our linked registry‐data, causes of death and diagnoses from in‐patient care were available until December 31, 2019. The following International Classification of Diseases (ICD) codes were used to define diagnoses: MI (ICD‐8/9 code 410; ICD‐10 code I21), ischemic heart disease (IHD) (ICD‐8/9 code 410–414, ICD‐10 code I20–I25), stroke (ICD‐8/9 code 431; 434; 436, ICD‐10 code I61; I63; except I63.6; I64), transient ischemic attack (TIA) (ICD‐8/9 code 435, ICD‐10 code G45.3; G45.9), and HF (ICD‐8/9 code 428, ICD‐10 code I50).

### Selection processes

Participants in VIP with at least one BP recorded in SSPS were eligible. For people with many SSPS recordings, the most recent BP before the date of the VIP‐examination was included. Participants with an SSPS‐BP recorded within 7 days prior to VIP were excluded, as these values were often identical to the VIP values and may represent VIP measurements entered into the electronic medical record.

In the analysis of agreement, participants with an SSPS‐BP recorded within 8–180 days before VIP were analyzed. One hundred eighty days were judged short enough to avoid long‐term BP trends but long enough to minimize selection bias from frequent visitors. Other time intervals were explored without important differences (Supporting Information). Because of the screening situation in VIP, individuals identified with high BP were often re‐measured in primary care shortly afterward. This created a regression toward the mean effect with lower subsequent SSPS‐BPs (Table S1). We therefore excluded SSPS‐BPs measured after VIP from further analyses.

For the CVD analyses, inclusion was expanded to VIP participants with SSPS‐BP recordings within 1.5 years before VIP to attain a sufficient number of participants and events. VIP‐examinations with missing systolic BP (SBP) and/or diastolic BP (DBP) were excluded. Participants with diagnosis of MI, IHD, stroke, TIA, or HF prior to the date of VIP‐examination were excluded. Individuals participating in several VIP‐examinations were included at the most recent visit before event or censoring.

### Statistical analysis

Comparing VIP‐ and SSPS‐BPs in the same person, mean differences, corresponding SDs, and 95% limits of agreement were calculated and plotted according to the Bland–Altman method, separately for SBP and DBP [18].

Subgroup analyses were performed according to sex, age category (<45, 45–<55, and ≥55 years), staff category performing the measurement in SSPS (doctor or “other”), prior diabetes, and hypertension diagnosis. Stratification was performed according to hypertensive status by each age category to assess potential interaction and according to calendar month of SSPS measurement to explore impact of seasonal variation in BP. Mean differences according to year of recording were explored as methods in VIP varied slightly over the years (Supporting Information).

Survival analyses were performed for VIP and SSPS‐BPs separately. Time at risk was calculated from the date of VIP‐examination until event or censoring. The primary outcome was a composite of first‐time hospitalization or death due to MI, stroke, or HF. Participants were censored at the end of the study period (December 31, 2019), on the date of death from other causes, or on 31 December of the last year prior to emigration.

Survival curves were produced by the Kaplan–Meier method. Cox proportional hazards regression was performed to estimate hazard ratios (HRs) with 95% confidence intervals (CIs). BP values were categorized according to 2025 American College of Cardiology/American Heart Association Guidelines classification [5]. Normal BP (<120/80 mmHg) was used as reference category. Cox regression was performed both unadjusted and adjusted for age, sex, total cholesterol, BMI, fasting glucose value, diabetes status, smoking status, educational level, civil status, and income. These were considered confounders for which sufficient data were available. Cases with missing data were dropped in the adjusted analysis. Sensitivity analysis with additional adjustment for the time period of the VIP‐recording was also performed (Supporting Information). Subgroup analyses were performed according to staff category, measuring the SSPS‐BP, and by prior hypertension diagnosis.

To visualize the continuous association between BP and CVD, restricted cubic splines with four knots were used in adjusted Cox regression [19]. Reference values were 110 and 70 mmHg in analysis of SBP and DBP, respectively. HRs were estimated at 10‐mmHg increments (120–160 mmHg for SBP, 80–100 mmHg for DBP). At each 10‐mmHg increment, the difference in log HRs from each measurement type was determined from 2000 bootstrap resamples, presented as the ratio of HRs with 95% CIs.

Population‐attributable fractions (PAFs) with 95% CI were calculated on the basis of adjusted Cox regression models with cutoffs corresponding to elevated BP, Stage 1, and Stage 2 hypertension, using Miettinen's formula [20].

Analyses were performed in IBM SPSS Statistics version 29.0.2.0 and R Statistical Software version 4.4.2.

## Results

### Analysis of agreement

A total of 9567 participants were included in the agreement analyses (Fig. S2). Their mean age was 53.3 (SD 7.6) years, 5393 (56.4%) were female, and 4043 (42.3%) had a previous hypertension diagnosis (Table S2). A doctor measured routine BP in 4959 (51.8%) cases. The median time between measurements was 84.0 (interquartile range 89.0) days.

Routine measurements were 4.0/1.7 mmHg higher compared to standardized measurements (Fig. 1). The limits of agreement ranged from −26.8 to 34.8 mmHg for SBP and from −17.9 to 21.2 mmHg for DBP. Very similar results were acquired independently of time frame for the routine measurement (Table S3).

**Fig. 1:**
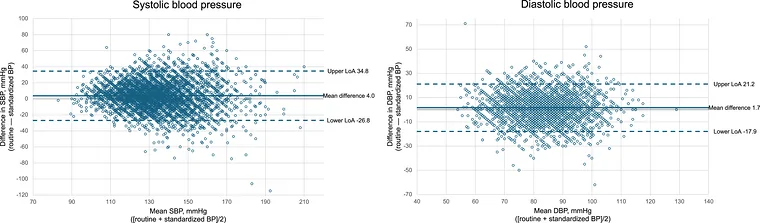
Bland–Altman plots for systolic and diastolic blood pressure, for routine BPs compared to standardized BPs. BP, blood pressure; DBP, diastolic blood pressure; LoA, limits of agreement; SBP, systolic blood pressure.

Subgroup analyses found some discrepancies in mean differences (Table 1). The most prominent difference was found for staff category, with 6.8/2.9 mmHg difference between routine and standardized BPs for doctors and 1.0/0.4 mmHg for other staff. Routine BPs were higher for participants without previous hypertension diagnosis (mean difference 5.4/2.4 mmHg) compared to those with hypertension (2.1/0.8 mmHg). Mean differences between routine and standardized BPs decreased as age increased, independent of hypertensive status (Table S4). Seasonal variation in BP had some impact on mean differences but was not a main determinant as routine BPs remained higher throughout the year (Table S5). Mean differences varied slightly by year of recording but were not clearly related to changes in measurement method for standardized BPs (Fig. S3).

**Table 1:** Mean differences and limits of agreement for standardized and routine blood pressures (BPs), according to subgroups.

				Systolic BP	Diastolic BP
Subgroup	*n* (%)	Mean routine BP, mmHg	Mean standardized BP, mmHg	Mean difference^a^ (95% LoA), mmHg	Mean difference^a^ (95% LoA), mmHg
Total	9567	135.4/83.3	131.4/81.6	4.0 (−26.8 to 34.8)	1.7 (−17.9 to 21.2)
Age category					
<45 years	1695 (17.7)	127.2/80.7	121.4/77.5	5.8 (−19.9 to 31.5)	3.1 (−16.2 to 22.5)
45–<55 years	3062 (32.0)	134.3/84.3	129.6/82.0	4.8 (−25.2 to 34.8)	2.3 (17.6–22.1)
≥55 years	4810 (50.3)	139.0/83.7	136.1/82.8	2.9 (−29.8 to 35.5)	0.8 (−18.5 to 20.1)
Sex					
Men	4174 (43.6)	136.4/84.7	133.1/83.7	3.3 (−26.3 to 32.9)	1.1 (−18.6 to 20.8)
Women	5393 (56.4)	134.7/82.2	130.1/80.1	4.6 (−27.0 to 36.1)	2.1 (−17.3 to 21.5)
Staff category recording routine BP					
Doctor	4959 (51.8)	135.9/83.0	129.1/80.1	6.8 (−23.9 to 37.6)	2.9 (−17.0 to 22.8)
Other	4608 (48.2)	134.9/83.7	133.9/83.3	1.0 (−28.7 to 30.6)	0.4 (−18.5 to 19.2)
Hypertensive status^b^					
Diagnosis	4043 (42.3)	140.0/86.2	137.9/85.4	2.1 (−30.6 to 34.8)	0.8 (−19.1 to 20.7)
No diagnosis	5524 (57.7)	132.1/81.2	126.7/78.8	5.4 (−23.5 to 34.3)	2.4 (−16.8 to 21.6)
Diabetes status^b^					
Diagnosis	672 (7.0)	136.3/82.5	134.9/82.2	1.4 (−29.4 to 32.2)	0.3 (−18.7 to 19.3)
No diagnosis	8895 (93.0)	135.4/83.4	131.2/81.6	4.2 (−26.5 to 34.9)	1.8 (−17.8 to 21.4)

Abbreviations: BP, blood pressure; LoA, limits of agreement.

^a^ Calculated as routine BP value (from Swedish Stroke Prevention Study)—standardized BP value (from Västerbotten Intervention Programme).

^b^ Defined as diagnosis present before the date of the routine measurement.

Routine measurements frequently misclassified BP into higher BP categories (Fig. 2). Overall, 41.4% of participants categorized as Stage 2 hypertension by routine BP had a lower BP when measured standardized. In total, 21.0% of participants with hypertension according to routine BP had non‐hypertensive standardized values. Only half as many had a normal BP according to routine measurements compared with standardized measurements. However, most people with normal routine BP remained “normal” when measured standardized.

**Fig. 2:**
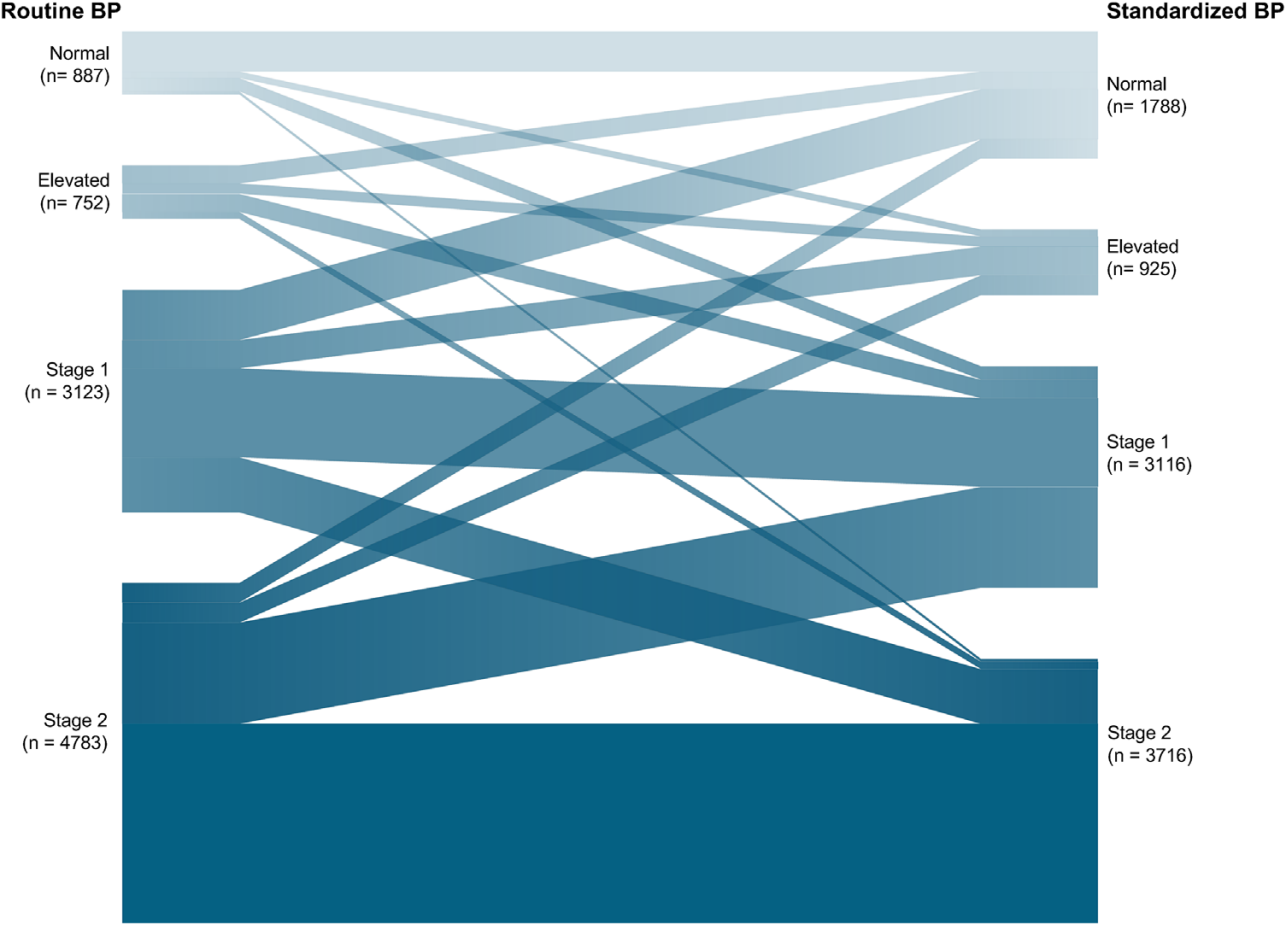
Sankey diagram of distribution of routine and standardized blood pressures according to categories: normal (<120/80 mmHg), elevated (120–129/<80 mmHg), Stage 1 hypertension (130–139/80–89 mmHg), and Stage 2 hypertension (≥140/90 mmHg). BP, blood pressure.

### Association with CVD

A total of 19,720 participants were included in the CVD analyses (Fig. S4). Participants closely resembled those in the agreement analysis (Table S6). During a total follow‐up time of 248,750 person‐years (mean 12.6 years per participant), 1910 participants experienced a CVD event, of which 36.9% were MI, 34.5% stroke, 22.5% HF, and 6.2% death from these causes. The mean time until event was 9.2 years.

Out of 4256 participants with normal BP according to the standardized measurement, 162 (3.8%) experienced a CVD event, compared to 96 out of 2120 (4.5%) of those with normal BP by the routine measurement (Fig. S5, Table S7).

CVD risk increased with increasing BP levels, to a higher degree for standardized measurements. Unadjusted HRs were 2.40 (95% CI 2.01–2.86) for Stage 1 hypertension and 4.04 (3.43–4.77) for Stage 2 hypertension for standardized measurements, and between 1.65 (1.33–2.05) and 2.69 (2.19–3.31), respectively, for routine BPs (Table S7).

In the adjusted analyses, 476 participants (2.4%) were excluded due to missing data for one or more covariates, which was considered negligible. The association between BP level and CVD was weaker in the adjusted analyses, but the relative differences between routine and standardized measurements remained approximately the same (Fig. 3). Elevated BP and Stage 1 hypertension entailed a 54% risk increase according to standardized BP but were not associated with increased risk for routine BPs. For Stage 2 hypertension, HR was 2.11 (95% CI 1.77–2.51) with standardized measurement and 1.43 (1.15–1.77) with routine measurement. Consistent results were seen in analyses of continuous BP, where routine BPs showed a non‐linear association with CVD (*p* = 0.03 for SBP, *p* = 0.04 for DBP), whereas standardized BPs displayed a near‐linear curve. The ratio of HRs ranged from 0.68 to 0.75 across all SBP levels, with consistently weaker association for routine measurements (Table S8, Fig. S6). Subgroup analyses stratified by hypertensive status or staff category measuring the routine BP could not explain the weaker association with CVD for routine BPs (Table S9). Additional adjustment for the time period when standardized BP was recorded did not meaningfully alter the results (Table S10).

**Fig. 3:**
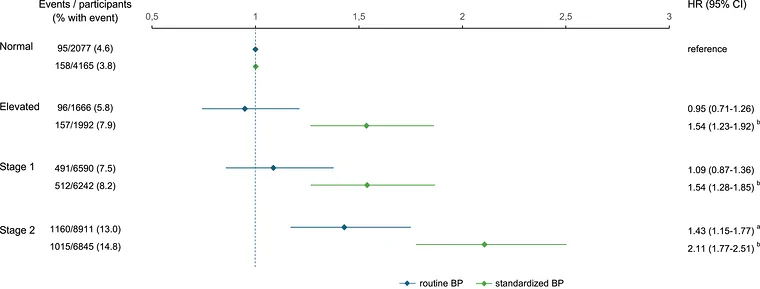
Association between BP category and cardiovascular disease for routine and standardized BPs, respectively. HRs are calculated by Cox regression adjusted for age, sex, BMI, total cholesterol, fasting glucose, diabetes, income, education level, civil status, and smoking status. Event is defined as first‐time hospitalization or death due to myocardial infarction, heart failure, or stroke. Normal <120/80 mmHg, elevated 120–129/<80 mmHg, Stage 1 hypertension 130–139/80–89 mmHg, and Stage 2 hypertension >140/90 mmHg. BP, blood pressure; CI, confidence interval; HR, hazard ratio. ^a^p < 0.05; ^b^p < 0.001.

Routine measurements were associated with a lower PAF for CVD events, despite including more people as exposed, compared with the standardized measurements (Fig. 4). PAF for BP levels above normal was 19.3% (95% CI 1.5–33.8) for routine measurements compared with 41.8% (32.0–50.1) for standardized measurements. PAF for Stage 2 hypertension was similar for routine and standardized measurements (18.3% and 21.3%, respectively), but 30% more were considered exposed with the routine BPs.

**Fig. 4:**
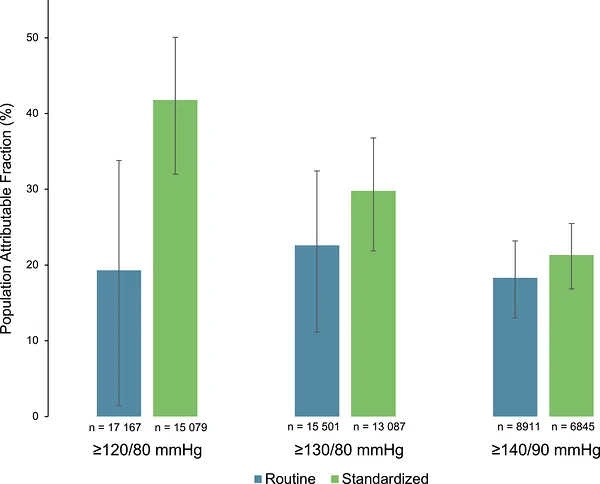
Population‐attributable fraction (PAF) with 95% confidence intervals for routine and standardized BPs at different BP levels. PAF is calculated based on HRs from adjusted Cox regressions, where reference is <120/80 mmHg, <130/80 mmHg, and <140/90 mmHg for each cutoff, respectively. BP, blood pressure.

## Discussion

This study compared routine BPs obtained in primary care with BPs measured according to a standardized protocol in the same individuals. Routine BPs differed from standardized BPs both in numeric agreement and in prognostic information. We found a systematic error where routine BPs were 4/2 mmHg higher compared to standardized measurements, but also random error was reflected in wide limits of agreement. This had a considerable impact on the classification of BP values into clinical categories and on the association between BP categories and risk for CVD. Using standardized measurements, twice as many were categorized as having normal BP compared to routine measurements. Elevated BP and Stage 1 hypertension implied a 54% risk increase with standardized measurements, whereas only Stage 2 hypertension was associated with CVD for routine measurements. With standardized measurements, elevated and hypertensive values together explained twice as many CVD cases compared with routine measurements, whereas fewer people were classified as exposed.

The difference in BP values between routine and standardized measurements shown here aligns with previous findings, although some studies have found an even greater difference [21, 22, 23, 24]. The magnitude of difference resembles the comparison to the “standard treatment” group in SPRINT, with fairly similar limits of agreement [12]. The systematic error differed slightly between subgroups. Routine BPs were closer to the standardized values in people with hypertension or diabetes; these BPs are more likely to be measured during follow‐up visits under calm circumstances resembling the standardized setting. Further, routine BPs were 6.8/2.9 mmHg higher than standardized BPs when measured by a physician, whereas the difference was only 1.0/0.4 mmHg for other staff categories. This is comparable to the findings from a meta‐analysis suggesting that doctors’ measurements are 7.0/3.8 mmHg higher than nurses [25]. In addition to the possible white coat phenomenon, it is plausible that physicians experience a lack of time to follow measurement recommendations to a larger extent and are more likely to record BP regardless of the reason for the visit, with possible influence from pain, stress, or acute illness.

To our knowledge, this is the first study comparing routine BPs to standardized BPs in terms of the association with CVD outcomes. Individuals were frequently misclassified according to their routine BP, thus diluting the association between hypertension and CVD. The misclassification of people as falsely hypertensive according to routine measurements is problematic because it may result in overdiagnosis and overtreatment. This is of particular interest in light of recent guidelines, lowering the threshold for antihypertensive treatment initiation to levels where the association between BP and CVD was only present for standardized measurements. Reassuringly, however, BP levels ≥140/90 mmHg, corresponding to the international definition of hypertension, were associated with increased cardiovascular risk regardless of measurement method; all major guidelines agree that such BP levels should be treated [4, 5, 6].

The discrepancy in prognostic value between standardized and routine BPs is further reflected in the estimated PAF for each method. Notably, PAFs are similar for BP values above 140 mmHg, but the total PAF for higher than normal BP is twice as large for standardized measurements due to the significant risk association in the 120–140/80–90 mmHg BP range.

The high variability and lower prognostic ability of routine BPs do not allow for simple translation into standardized BPs, as done for out‐of‐office BPs in major guidelines [4, 5, 6]. Several guidelines have suggested increased use of home‐ and ambulatory BP measurement, as these methods better predict CVD than office BP measurement [26, 27, 28]. However, there is to date no definitive evidence from clinical outcome trials using home‐ or ambulatory BP measurement to guide treatment, as existing trials have been inconclusive or limited to surrogate outcomes [29, 30]. Unattended automated office BP measurement may reduce white‐coat effect and ensure a quiet environment and multiple readings, but similarly, treatment thresholds and targets remain uncertain for this method as well [31]. Improving the quality of clinical BP measurements is the evident call for action, as the application of current treatment thresholds and targets, derived from highly standardized clinical trial settings, is questionable in routine care today, based on our findings.

### Limitations

This study is limited to a middle‐aged Western European population in a primary care setting; extrapolation to other regions, age groups, and health care settings should be made with caution. Further, automatic devices, unattended office BP measurements, and home BP measurements are probably more frequently used today than in the study sample, as it is based on BPs measured before 2012. Importantly, however, due to the time lag since the last BP recording, we have been able to assess CVD outcomes, adding to the literature on the prognostic relevance of BP measurements in different settings.

Another limitation is the time lag between the compared BPs. Ideally, two measurements compared should be recorded as close to each other as possible to reduce changes in BP from other causes. With an observational design, however, BPs are not available, and shorter time intervals also introduce selection bias related to underlying reasons for BP measurement. Six months, as applied here, are somewhat arbitrary but should be considered a compromise between avoiding changes in BP and avoiding selection bias of frequent visitors in primary care. Reassuringly, results were unaltered in sensitivity analyses assessing different time intervals.

Theoretically, differences between measurement methods in association with CVD could be a result of residual confounding. However, including the most important confounders, the association between BP category and CVD was weakened, but the relative difference between measurement types for the same BP category remained approximately the same. Moreover, the matched design, comparing the same individuals classified by different measurement methods, also makes this unlikely.

Lastly, different validated automated measurement devices may have been used across centers for the standardized measurements, potentially introducing some variability in standardized BPs. Additional information on routine measurement procedure, antihypertensive treatment, and reason for primary care visit would have been useful to further assess the causes of the systematic differences and variability observed. Future studies should include such information and explore this further.

## Conclusion

Routine clinical BPs differ from standardized measurements in a systematic and random manner, resulting in substantial misclassification and an attenuated association with CVD. Given that routine measurements commonly overestimate BP, and the lack of association between routine BP levels and CVD <140/90 mmHg, applying guideline recommendations derived from clinical trials employing highly standardized protocols may be questionable in routine care. To ensure adequate treatment and management of hypertension, the quality of BP measurement should be improved.

## Author contributions

**Olivia Gästgivars**: Conceptualization; data curation; formal analysis; methodology; visualization; writing—original draft; writing—review and editing. **Joachim Ögren**: Conceptualization; methodology; supervision; writing—review and editing. **Bo Carlberg**: Conceptualization; methodology; supervision; writing—review and editing. **Mattias Brunström**: Conceptualization; methodology; data curation; funding acquisition; supervision; visualization; writing—original draft; writing—review and editing; project administration.

## Conflict of interest statement

M.B. has received modest honoraria from AstraZeneca, Medtronic, and Amarin and is an executive officer for the European Society of Hypertension. All other authors declare no conflicts of interest.

## Funding information

Umeå University and Västerbotten county council (ALF, grant number RV‐1010705, RV‐1005575), the Swedish Heart and Lung Foundation (grant number 20241373), and the Heart Foundation of Northern Sweden.

## Ethics statement

The study was approved by the Swedish Ethical Review Authority (dnr 2020–04149).

## Supporting information




**Table S1**: Mean differences and limits of agreement for VIP and SSPS values, recorded after VIP.
**Table S2**: Characteristics of participants in VIP, with one preceding BP‐measurement in SSPS within 180 days.
**Table S3**: Mean differences and limits of agreement for standardized and routine BP values recorded before the standardized.
**Table S4**: Mean differences according to hypertensive status for different age categories.
**Table S5**: Mean differences and limits of agreement for standardized and routine BP values, according to calendar month.
**Table S6**: Characteristics of participants in VIP, with one preceding BP‐measurement in SSPS within 1.5 years.
**Table S7**: Association between blood pressure level and cardiovascular disease, unadjusted analysis.
**Table S8**: Association between blood pressure and cardiovascular disease in continuous analyses.
**Table S9**: Association with cardiovascular disease, by subgroups of hypertensive status and staff measuring routine BP.
**Table S10**: Association with cardiovascular disease, additionally adjusted for time period when standardized blood pressure was recorded.
**Figure S1**: Timeline of included registers and cohorts.
**Figure S2**: Flowchart of selection for the analysis of agreement.
**Figure S3**: Mean blood pressure and mean differences according to year of blood pressure recording.
**Figure S4**: Flowchart of selection for the analysis of association to CVD.
**Figure S5**: Kaplan–Meier curves and number at risk tables for survival probability of cardiovascular disease according to blood pressure category.
**Figure S6**: Association between blood pressure and cardiovascular disease, continuous analyses.
Supporting Information: Agreement analyses according to different timepoints of routine blood pressures. Sensitivity analyses according to year of blood pressure recording.
**Supporting Information**: Sensitivity analyses according to year of blood pressure recording.

## Data Availability

The data used in this study is protected by legislation and hence not publicly available. The data can, however, be handed out on reasonable request for research purposes (e‐mail: olivia.gastgivars@umu.se).
