# Supplementary material for: Routine versus standardized blood pressure measurements: Agreement and association with cardiovascular disease
Source: J Intern Med. 2026 Sep 24;300(6):821–31. doi: 10.1111/joim.70164 (PMC13741809; doi:10.1111/joim.70164)
Supplement: Supplementary file 1 [file JOIM-300-821-s001.docx]

**Supporting Information**

**Table S1**. Mean differences and limits of agreement for VIP and SSPS values, recorded after VIP.

**Table S2**. Characteristics of participants in VIP, with one preceding BP-measurement in SSPS within 180 days.

**Table S3.** Mean differences and limits of agreement for standardized and routine BP values recorded before the standardized.

**Table S4**. Mean differences according to hypertensive status, for different age categories.

**Table S5**. Mean differences and limits of agreement for standardized and routine BP values, according to calendar month.

**Table S6**. Characteristics of participants in VIP, with one preceding BP-measurement in SSPS within 1,5 years.

**Table S7**. Association between blood pressure level and cardiovascular disease, unadjusted analysis.

**Table S8.** Association between blood pressure and cardiovascular disease in continuous analyses.

**Table S9**. Association with cardiovascular disease, by subgroups of hypertensive status and staff measuring routine BP.

**Table S10**. Association with cardiovascular disease, additionally adjusted for time period when standardized blood pressure was recorded.

**Figure S1**. Timeline of included registers and cohorts.

**Figure S2**. Flowchart of selection for the analysis of agreement.

**Figure S3.** Mean blood pressure and mean differences according to year of blood pressure recording.

**Figure S4**. Flowchart of selection for the analysis of association to CVD.

**Figure S5.** Kaplan-Meier curves and number at risk tables, for survival probability of cardiovascular disease according to blood pressure category.

**Figure S6**. Association between blood pressure and cardiovascular disease, continuous analyses.

**Supplementary Methods**

Agreement analyses according to different timepoints of routine blood pressures

Sensitivity analyses according to year of blood pressure recording

**Supplementary Results**

Sensitivity analyses according to year of blood pressure recording

**Table S1**. Mean differences and limits of agreement for VIP and SSPS values, recorded after VIP.

| **Time interval for SSPS-BP,**  days after VIP | |  |  | | **Systolic BP** | **Diastolic BP** | | |
| --- | --- | --- | --- | --- | --- | --- | --- | --- |
|  |  | **n** | **Mean SSPS BP**, mmHg | **Mean VIP BP**, mmHg | **Mean difference ^a^ (95% LoA),** mmHg | **Mean difference ^a^ (95% LoA),** mmHg | | |
|  | |  | |  |  |  |  |  |
|  | 30 days | 8680 | 136.0/85.0 | 138.0/86.1 | -2.0 (-25.3 to 21.4) | -1.2 (-16.0 to 13.6) | | |
|  | 90 days | 13 075 | 136.3/84.8 | 137.2/85.5 | -0.9 (-28.8 to 25.0) | -0.6 (-16.9 to 15.6) | | |
|  | 180 days | 16 806 | 136.2/84.5 | 135.8/84.5 | 0.4 (-26.9 to 27.6) | 0.0 (-17.2 to 17.2) | | |
|  | 365 days | 23 327 | 136.0/84.2 | 134.1/83.3 | 1.9 (-26.8 to 30.6) | 0.9 (-17.1 to 19.0) | | |
|  | |  |  |  |  |  | | |

BP = blood pressure; SSPS = Swedish Stroke Prevention Study; VIP = Västerbotten Intervention Programme; LoA = Limits of agreement

^a^ Calculated as SSPS BP – VIP BP.

**Table S2.** Characteristics of participants in VIP, with one preceding BP-measurement in SSPS within 180 days.

| **Baseline characteristics** | **n (%)** | **n missing (%)** |
| --- | --- | --- |
| Gender |  |  |
| Female | 5393 (56.4) |  |
| Male | 4174 (43.6) |  |
| Hypertension ^a^ | 4043 (42.3) |  |
| Diabetes ^a^ | 672 (7.0) |  |
| Heart failure ^a^ | 54 (0.6) |  |
| Stroke ^a^ | 156 (1.6) |  |
| Myocardial infarction ^a^ | 248 (2.6) |  |
| Smoking status |  | 146 (1.5) |
| Non-smoker | 4405 (46.0) |  |
| Smoker | 1300 (13.6) |  |
| Ex-smoker | 2603 (27.2) |  |
| Occasional smoker | 198 (2.1) |  |
| Previously occasional | 915 (9.6) |  |
| Marital status |  | 7 (0.1) |
| Not married | 1975 (20.6) |  |
| Married | 5901 (61.7) |  |
| Divorced | 1415 (14.8) |  |
| Widowed | 269 (2.8) |  |
| Educational level |  | 23 (0.2) |
| Less than high school | 1751 (18.3) |  |
| High school degree | 4954 (51.8) |  |
| College degree or higher | 2839 (29.7) |  |
|  | **Mean (SD)** | **n missing (%)** |
| Age | 53.3 (7.6) |  |
| BMI, *kg/m^2^* | 27.6 (4.9) | 25 (0.3) |
| Total cholesterol, *mmol/l* | 5.5 (1.1) | 29 (0.3) |
| Fasting glucose, *mmol/l* | 5.8 (1.5) | 47 (0.5) |
| Income decile | 5.7 (2.9) | 7 (0.1) |
| Routine BP (SSPS) |  |  |
| Systolic, *mmHg* | 135.4 (16.7) |  |
| Diastolic, *mmHg* | 83.3 (9.7) |  |
| Standardized BP (VIP) |  |  |
| Systolic, *mmHg* | 131.4 (17.5) | 19 (0.2) |
| Diastolic, *mmHg* | 81.6 (10.4) | 29 (0.3) |
|  | **Median (IQR)** |  |
| Time between routine and standardized BP, *days* | 84.0 (89.0) |  |

BMI = body mass index; SSPS = Swedish Stroke Prevention Study; VIP = Västerbotten Intervention Programme; BP = blood pressure; IQR = Interquartile range

^a^ Diagnosis before date of the routine BP measurement (SSPS).

**Table S3**. Mean differences and limits of agreement for standardized and routine BP values recorded before the standardized.

| **Time interval for routine BP,**  days before standardized BP | |  |  | | **Systolic BP** | **Diastolic BP** | | |
| --- | --- | --- | --- | --- | --- | --- | --- | --- |
|  |  | **n** | **Mean routine BP,** mmHg | **Mean standardized BP,** mmHg | **Mean difference ^a^ (95% LoA),** mmHg | **Mean difference ^a^ (95% LoA),** mmHg | | |
|  | |  | |  |  |  |  |  |
|  | 30 days | 1678 | 138.0/84.7 | 133.1/82.8 | 4.9 (-24.8 to 34.6) | 1.9 (-16.6 to 20.3) | | |
|  | 90 days | 5163 | 136.2/83.7 | 132.0/81.9 | 4.2 (-26.3 to 34.8) | 1.8 (-17.4 to 21.0) | | |
|  | 180 days | 9567 | 135.4/83.3 | 131.4/81.6 | 4.0 (-26.8 to 34.8) | 1.7 (-17.9 to 21.2) | | |
|  | 365 days | 17 148 | 134.7/82.9 | 130.7/81.3 | 4.0 (-26.9 to 34.8) | 1.7 (-18.0 to 21.4) | | |
|  | |  |  |  |  |  | | |

BP = blood pressure; LoA = Limits of agreement

^a^ Calculated as routine BP value (from Swedish Stroke Prevention Study) – standardized BP value (from Västerbotten Intervention Programme).

**Table S4**. Mean differences according to hypertensive status, for different age categories.

| **Subgroup** | | **< 45-year-olds** | | **45 to <55-year-olds** | | **≥ 55-year-olds** | |
| --- | --- | --- | --- | --- | --- | --- | --- |
|  |  | **n (%)** | **Systolic/diastolic mean difference ^a^,** mmHg | **n (%)** | **Systolic/diastolic mean difference ^a^,**  mmHg | **n (%)** | **Systolic/diastolic mean difference ^a^,**  mmHg |
| Total | | 1695 | 5.8/3.1 | 3062 | 4.8/2.3 | 4810 | 2.9/0.8 |
| Hypertensive status^b^ | |  |  |  |  |  |  |
|  | Diagnosis | 293 (17.3) | 4.1/2.4 | 1182 (38.) | 2.8/1.2 | 2567 (53.4) | 1.5/0.4 |
|  | No diagnosis | 1402 (82.7) | 6.1/3.3 | 1880 (61.4) | 6.1/3.0 | 2243 (46.6) | 4.4/1.3 |
|  | |  |  |  |  |  |  |

BP = blood pressure

^a^ Calculated as routine BP value (from Swedish Stroke Prevention Study) – standardized BP value (from Västerbotten Intervention Programme).

^b^ Defined as diagnosis present before the date of the routine measurement.

**Table S5**. Mean differences and limits of agreement for standardized and routine BP values, according to calendar month.

| **Calendar month for routine measurement** | |  |  | | **Systolic BP** | **Diastolic BP** | | |
| --- | --- | --- | --- | --- | --- | --- | --- | --- |
|  |  | **n** | **Mean routine BP,** mmHg | **Mean standardized BP,** mmHg | **Mean difference ^a^ (95% LoA),** mmHg | **Mean difference ^a^ (95% LoA),** mmHg | | |
|  | |  | |  |  |  |  |  |
|  | January | 935 | 136.5/83.5 | 132.0/81.6 | 4.4 (-26.2 to 35.0) | 1.9 (-17.9 to 21.6) | | |
|  | February | 693 | 137.6/84.1 | 132.6/82.0 | 4.9 (-25.5 to 35.4) | 2.1 (-17.3 to 21.5) | | |
|  | March | 698 | 136.5/84.2 | 131.6/82.4 | 5.0 (-23.7 to 33.6) | 1.8 (-16.2 to 19.8) | | |
|  | April | 610 | 135.0/83.1 | 131.9/81.7 | 3.2 (-26.8 to 33.1) | 1.3 (-19.2 to 21.8) | | |
|  | May | 639 | 133.9/82.6 | 131.3/81.7 | 2.6 (-27.3 to 32.6) | 1.0 (-18.0 to 20.0) | | |
|  | June | 615 | 134.0/83.0 | 131.8/81.5 | 2.2 (-28.4 to 32.8) | 1.5 (-19.0 to 22.0) | | |
|  | July | 388 | 133.6/83.1 | 131.8/81.7 | 2.0 (-28.1 to 32.1) | 1.4 (-18.2 to 21.0) | | |
|  | August | 772 | 134.6/82.7 | 130.7/81.1 | 3.9 (-28.5 to 36.4) | 1.5 (-17.3 to 20.4) | | |
|  | September | 1065 | 136.0/83.6 | 131.2/81.7 | 4.8 (-24.8 to 34.3) | 1.9 (-17.5 to 21.3) | | |
|  | October | 1122 | 135.8/83.5 | 130.9/81.2 | 4.8 (-25.9 to 35.6) | 2.2 (-17.6 to 22.0) | | |
|  | November | 1083 | 134.5/82.9 | 130.7/81.5 | 3.8 (-28.8 to 36.3) | 1.5 (-18.3 to 21.2) | | |
|  | December | 928 | 135.6/83.3 | 131.5/81.7 | 4.1 (-27.4 to 35.7) | 1.6 (-18.3 to 21.6) | | |
|  | |  |  |  |  |  | | |

BP = blood pressure; LoA = Limits of agreement

^a^ Calculated as routine BP value (from Swedish Stroke Prevention Study) – standardized BP value (from Västerbotten Intervention Programme).

**Table S6.** Characteristics of participants in VIP, with one preceding BP-measurement in SSPS within 1,5 years.

| **Baseline characteristics** | **n (%)** | **n missing (%)** |
| --- | --- | --- |
| Gender |  |  |
| Female | 11 461 (58.1) |  |
| Male | 8259 (41.9) |  |
| Hypertension ^a^ | 7083 (35.9) |  |
| Diabetes ^a^ | 1037 (5.3) |  |
| Smoking status |  | 295 (1.5) |
| Non-smoker | 9307 (47.2) |  |
| Smoker | 2722 (13.8) |  |
| Ex-smoker | 4942 (25.1) |  |
| Occasional smoker | 499 (2.5) |  |
| Previously occasional | 1955 (9.9) |  |
| Marital status |  | 15 (0.1) |
| Not married | 4310 (21.9) |  |
| Married | 12 028 (61.0) |  |
| Divorced | 2820 (14.3) |  |
| Widowed | 547 (2.8) |  |
| Educational level |  | 49 (0.2) |
| Less than high school | 3216 (16.3) |  |
| High school degree | 10 179 (51.6) |  |
| College degree or higher | 6276 (31.8) |  |
|  | **Mean (SD)** | **n missing (%)** |
| Age | 52.8 (7.9) |  |
| BMI, kg/m^2^ | 27.3 (4.8) | 36 (0.2) |
| Total cholesterol, mmol/l | 5.5 (1.1) | 52 (0.3) |
| Fasting glucose, mmol/l | 5.7 (1.3) | 55 (0.3) |
| Income decile | 5.6 (2.9) | 15 (0.1) |
| Routine BP (SSPS) |  |  |
| Systolic, mmHg | 133.9 (16.3) |  |
| Diastolic, mmHg | 82.6 (9.6) |  |
| Standardized BP (VIP) |  |  |
| Systolic, mmHg | 130.0 (17.6) |  |
| Diastolic, mmHg | 81.0 (10.5) |  |
|  | **Median (IQR)** |  |
| Time between routine and standardized BP, *days* | 222.0 (263) |  |

BMI = Body mass index; SSPS = Swedish Stroke Prevention Study; VIP = Västerbotten Intervention Programme; BP = Blood pressure; SD = Standard deviation; IQR = Interquartile range

^a^ Diagnosis before date of the standardized measurement.

**Table S7.** Association between blood pressure level and cardiovascular disease, unadjusted analysis.

| **BP-category ^b^** | **Routine BP (SSPS)** | | | **Standardized BP (VIP)** | | | |
| --- | --- | --- | --- | --- | --- | --- | --- |
|  | **Cardiovascular events ^a^ / total number of observations (% with event)** | **HR (95% CI)** | **p-value** | | **Cardiovascular events ^a^ / total number of observations (% with event)** | **HR (95% CI)** | **p-value** |
| Normal | 96/2120 (4.5) | 1 (ref) |  | 162/4256 (3.8) | | 1 (ref) |  |
| Elevated | 102/1713 (6.0) | 1.28 (0.97-1.69) | 0.084 | 159/2040 (7.8) | | 2.04 (1.64-2.54) | <0.001 |
| Stage 1 | 508/6750 (7.5) | 1.65 (1.33-2.05) | <0.001 | 529/6397 (8.3) | | 2.40 (2.01-2.86) | <0.001 |
| Stage 2 | 1204/9137 (13.2) | 2.69 (2.19-3.31) | <0.001 | 1060/7027 (15.1) | | 4.04 (3.43-4.77) | <0.001 |

BP = blood pressure, SSPS = Swedish Stroke Prevention Study, VIP = Västerbotten Intervention Programme, HR = hazard ratio, CI = confidence interval.

^a^ First hospitalization or death due to myocardial infarction, stroke, heart failure.

^b^ Normal <120/80 mmHg, elevated 120-129/<80 mmHg, stage 1 hypertension 130-139/80-89 mmHg, stage 2 hypertension >140/90 mmHg

**Table S8.** Association between blood pressure and cardiovascular disease in continuous analyses.

|  | **Routine BP (SSPS)** | **Standardized BP (VIP)** | Routine/standardized HR ratio (95% CI) |
| --- | --- | --- | --- |
| **Systolic BP** | HR (95% CI) | HR (95% CI) |  |
| 110 mmHg | 1 (ref) | 1 (ref) |  |
| 120 mmHg | 0.95 (0.83-1.08) | 1.26 (1.12-1.43) | 0.75 (0.64-0.87) |
| 130 mmHg | 1.01 (0.84-1.22) | 1.49 (1.30-1.72) | 0.68 (0.56-0.83) |
| 140 mmHg | 1.24 (1.04-1.47) | 1.70 (1.46-1.98) | 0.73 (0.61-0.88) |
| 150 mmHg | 1.44 (1.21-1.71) | 1.92 (1.65-2.25) | 0.75 (0.63-0.90) |
| 160 mmHg | 1.59 (1.33-1.89) | 2.16 (1.85-2.53) | 0.73 (0.61-0.88) |
| **Diastolic BP** |  |  |  |
| 70 mmHg | 1 (ref) | 1 (ref) |  |
| 80 mmHg | 1.01 (0.91-1.13) | 1.12 (1.03-1.22) | 0.90 (0.79-1.03) |
| 90 mmHg | 1.22 (1.07-1.40) | 1.33 (1.16-1.51) | 0.92 (0.79-1.08) |
| 100 mmHg | 1.48 (1.28-1.72) | 1.70 (1.48-1.95) | 0.87 (0.75-1.02) |

Estimated HRs determined from adjusted Cox regression where blood pressure was modeled continuously with restricted cubic spline function. The outcome was first hospitalization or death due to myocardial infarction, stroke, heart failure. BP = blood pressure, SSPS = Swedish Stroke Prevention Study, VIP = Västerbotten Intervention Programme, HR = hazard ratio, CI = confidence interval.

**Table S9.** Association with cardiovascular disease ^a^, by subgroups of hypertensive status and staff measuring routine BP.

|  | **Subgroup, BP-category ^b^** | | | **Routine BP (SSPS)** | | **Standardized BP (VIP)** | |
| --- | --- | --- | --- | --- | --- | --- | --- |
|  | **Hypertensive status** | | | HR (95% CI) | p-value | HR (95% CI) | p-value |
|  | Hypertension diagnosis  *(n = 6808, n events = 887 [13.0%])* | | | | | | |
|  | | Normal | | 1 (ref) |  | 1 (ref) |  |
|  | | Elevated | | 0.52 (0.30-0.90) | 0.02 | 1.11 (0.68-1.80) | 0.68 |
|  | | Stage 1 | | 0.70 (0.47-1.04) | 0.08 | 1.19 (0.79-1.79) | 0.40 |
|  | | Stage 2 | | 0.85 (0.58-1.25) | 0.41 | 1.53 (1.03-2.28) | 0.03 |
| No hypertension diagnosis  *(n = 12 436, n events = 955 [7.7%])* | | | | | | | |
|  | | Normal | | 1 (ref) |  | 1 (ref) |  |
|  | | Elevated | | 1.10 (0.79-1.54) | 0.57 | 1.57 (1.22-2.02) | <0.001 |
|  | | Stage 1 | | 1.11 (0.85-1.45) | 0.46 | 1.45 (1.18-1.79) | <0.001 |
|  | | Stage 2 | | 1.47 (1.14-1.91) | 0.003 | 1.94 (1.58-2.39) | <0.001 |
|  | **Staff measuring routine BP** | |  | | | | |
|  | Doctor  *(n = 10 345, n events = 1022 [9.9%])* | | | | | | |
|  | | Normal | | 1 (ref) |  | 1 (ref) |  |
|  | | Elevated | | 0.94 (0.63-1.42) | 0.78 | 1.46 (1.12-1.91) | 0.005 |
|  | | Stage 1 | | 1.22 (0.89-1.67) | 0.217 | 1.45 (1.16-1.80) | 0.001 |
|  | | Stage 2 | | 1.51 (1.11-2.05) | 0.009 | 2.00 (1.62-2.48) | <0.001 |
|  | Other  *(n = 8899, n events = 820 [9.2%])* | | |  |  |  |  |
|  | | Normal | | 1 (ref) |  | 1 (ref) |  |
|  | | Elevated | | 0.96 (0.64-1.42) | 0.82 | 1.78 (1.18-2.70) | 0.006 |
|  | | Stage 1 | | 0.95 (0.70-1.30) | 0.76 | 1.84 (1.30-2.59) | <0.001 |
|  | | Stage 2 | | 1.35 (1.00-1.82) | 0.05 | 2.52 (1.80-3.52) | <0.001 |
|  |  | | |  |  |  |  |

Cox regression adjusted for age, sex, BMI, total cholesterol, fasting glucose, diabetes, income, education level, civil status, smoking status.

BP = blood pressure, SSPS = Swedish Stroke Prevention Study, VIP = Västerbotten Intervention Programme, HR = hazard ratio, CI = confidence interval.

^a^ First hospitalization or death due to myocardial infarction, stroke, heart failure.

^b^ Normal <120/80 mmHg, elevated 120-129/<80 mmHg, stage 1 hypertension 130-139/80-89 mmHg, stage 2 hypertension >140/90 mmHg

**Table S10.** Association with cardiovascular disease ^a^, additionally adjusted for time period when standardized blood pressure was recorded.

| **BP-category**^b^ | **Routine BP (SSPS)** | | **Standardized BP (VIP)** | | |
| --- | --- | --- | --- | --- | --- |
|  | HR (95% CI) | p-value | | HR (95% CI) | p-value |
| Normal | 1 (ref) |  | | 1 (ref) |  |
| Elevated | 0.94 (0.71-1.25) | 0.67 | | 1.52 (1.22-1.90) | <0.001 |
| Stage 1 | 1.08 (0.87-1.35) | 0.49 | | 1.54 (1.29-1.85) | <0.001 |
| Stage 2 | 1.39 (1.12-1.72) | <0.05 | | 2.06 (1.73-2.46) | <0.001 |

Cox regression adjusted for age, sex, BMI, total cholesterol, fasting glucose, diabetes, income, education level, civil status, smoking status, and time period when standardized BP was recorded.

BP = blood pressure, SSPS = Swedish Stroke Prevention Study, VIP = Västerbotten Intervention Programme, HR = hazard ratio, CI = confidence interval.

^a^ First hospitalization or death due to myocardial infarction, stroke, heart failure.

^b^ Normal <120/80 mmHg, elevated 120-129/<80 mmHg, stage 1 hypertension 130-139/80-89 mmHg, stage 2 hypertension >140/90 mmHg

**Figure S1.** Timeline of included registers and cohorts.

BP = Blood pressure; SSPS = Swedish Stroke Prevention Study; VIP = Västerbotten Intervention Programme; NPR = National Patient Register; SCDR = Swedish Cause of Death Register

**Figure S2.** Flowchart of selection for the analysis of agreement between routine BPs (from SSPS) and standardized measurements (from VIP).

VIP = Västerbotten Intervention Programme; SSPS = Swedish Stroke Prevention Study; BP = blood pressure; SBP = systolic blood pressure; DBP = diastolic blood pressure.

**Figure S3.** Mean blood pressure and mean differences according to year of blood pressure recording.

To the left, mean systolic BP according to year of BP recording for routine (SSPS) and standardized BP (VIP), respectively. To the right, mean differences between routine (SSPS) and standardized BP (VIP) according to year of BP recording.

BP = blood pressure; SSPS = Swedish Stroke Prevention Study; VIP = Västerbotten Intervention Programme.

**Figure S4.** Flowchart of selection for the analysis of association to CVD.

VIP = Västerbotten Intervention Programme; SBP = systolic blood pressure; DBP = diastolic blood pressure; SSPS = Swedish Stroke Prevention Study; CVD = cardiovascular disease.

**Figure S5.** Kaplan-Meier curves and number at risk tables, for survival probability of cardiovascular disease according to blood pressure category.

To the left, routine blood pressures (from Swedish Stroke Prevention Study) and to the right, standardized blood pressure measurements (from Västerbotten Intervention Programme). Normal <120/80 mmHg, elevated 120–129/<80 mmHg, stage 1 hypertension 130-139/80-89 mmHg, stage 2 hypertension >140/90 mmHg.

**Figure S6.** Association between blood pressure and cardiovascular disease, continuous analyses.

Hazard ratios with corresponding confidence intervals (CI) for systolic and diastolic blood pressure, from adjusted Cox regression where blood pressure was modeled using restricted cubic splines, for routine and standardized blood pressure respectively. The outcome was first hospitalization or death due to myocardial infarction, stroke, heart failure. Spline knots are represented by dots on each curve.

**Supplementary Methods**

**Agreement analyses according to different timepoints of routine blood pressures**

Participants from Västerbotten Intervention Programme (VIP) with at least one blood pressure (BP) recorded in Swedish Stroke Prevention Study (SSPS) were included. The most recent and/or the next upcoming BP recording from SSPS was included in the dataset. Participants with SSPS-BPs measured within 7 days prior to or 7 days after VIP were excluded as they might represent VIP values entered into the electronic medical record. Agreement analyses were tested for participants with a SSPS-BP measured within 30, 90 and 365 days before and within 30, 90, 180 and 365 days after VIP. Agreement analyses were performed according to the Bland-Altman method as in the main analysis.

**Sensitivity analyses according to year of blood pressure recording**

Methods for BP measurement in VIP varied slightly over the years. Automated devices were introduced from 2000 onwards, and BP was measured seated from 2009-09-01 [1]. Because of these variations, differences between BP from SSPS and from VIP by year and time-period were investigated.

**Agreement analyses stratified by year**

Mean differences (SSPS – VIP-BP) with SDs were calculated. Most adjacent SSPS-BP to VIP measured within 180 days before VIP was used. Mean BP in SSPS and VIP were calculated according to year.

**Cox regression adjusted for time period of measurement**

Cox regression was performed, as in the main analysis (adjusted for age, sex, total cholesterol, BMI, fasting glucose value, diabetes status, smoking status, educational level, civil status, and income) and additionally adjusted for time period of VIP-examination (before 2000-01-01, between 2000-01-01 and 2009-09-01, and after 2009-09-01).

**Supplementary Results**

**Sensitivity analyses according to year of blood pressure recording**

Measurements before 2000-01-01

Mean difference was 5.8/1.6 mmHg (SD 15.7/9.6) for n = 1406. 75.8% of the measurements in SSPS were performed by a doctor. The mean BP in SSPS was 140.2/84.5 mmHg and 134.5/82.9 mmHg in VIP.

Measurements between 2000-01-01 and 2009-09-01

The mean difference was 3.4/1.9 mmHg (SD 15.8/9.9) for n = 6039. 47.4% of the measurements in SSPS were performed by a doctor. The mean BP in SSPS was 135.3/83.0 mmHg and 131.9/81.1 mmHg in VIP.

Measurements after 2009-09-01

Mean difference was 4.5/1.3 mmHg (SD 15.4/10.4) for n = 2120. 48.5% of the measurements in SSPS were performed by a doctor. The mean BP in SSPS was 132.5/83.6 mmHg and 128.0/82.3 mmHg in VIP.

Mean differences were larger before 2000. Before 2000, sample sizes each year were smaller and mean BPs in both SSPS and VIP higher, entailing higher degree of uncertainty and more variability. There were also a higher proportion of SSPS-BPs measured by doctors before 2000. Overall, the trends in mean difference were considered relatively stable from 2000 onwards.
